# Quackery, and a Suggestion

**Published:** 1900-03

**Authors:** 


### *EDITORIAL NOTES AND COMMENTS.*

The Business office of THE JOURNAL-RECORD is 305, 306 Fitten Building.

The Editorial office is 318-319-320 The Prudential.

Address all Business communications to Dr. M. B. Hutchins, Mgr.

Make remittances payable to THE ATLANTA JOURNAL-RECORD OF MEDICINE.

On matters pertaining to the Editorial and Original Communications address Dr. Bernard Wolff, Atlanta.

Reprints of original articles will be furnished at cost price. Requests for the same should always be made *on the manuscript.*

We will present, post-paid, on request, to each contributor of an original article, twenty (20) marked copies of THE JOURNAL-RECORD containing such article.

### QUACKERY, AND A SUGGESTION.

IT is not necessary to announce that quackery is rampant in Georgia, and especially in Atlanta. The fact is sufficiently advertised in newspapers and may be read on the run. Atlanta has quite a reputation to sustain as a snug harbor for medical derelicts, and as the time and laws pass, she does not block her port.

But whose fault is it? Where does the blame lie? We have a State Board of Medical Examiners vested with plenary powers as regards the test of fitness by examination of applicants for license to practice medicine in the State. Its function is educational, not police. Back of the Board of Examiners is the buttress of the law, which is satisfactory enough in the observance, but more honored in the breech. We, have then, the means at hand for cleaning the State of the filth of charlatanism, or at least of preventing further deposits. Why do not we avail ourselves of these means ready at hand?

It is because the medical profess.on is a great, hulking, silent altruistic booby that must needs have some one come in when it has bemerded itself and change its diaper. It does not even know when it is dirty. It has neither the pride nor the self-respect nor the energy to look after its simplest, most fundamental interests. It allows itself, prone, inert, an Ally Sloper grin on its blank face, to be stepped on and derided by every Weary Raggles of a medical hobo who has the price of a half-column newspaper ad. in his pocket. It giggles and says: "You behave," and acquiesces when it is chucked under the chin by any whore of Babylon who wants to coax "testimonial" and "prominent physician" endorsements out of it. We are no more capable of self-government than are the Filipinos or the Haytians, or others who have to hire other people to do their thinking. It is useless for gentlemen of bulging foreheads to attempt to deny it. We cannot uproot the quacks by ourselves; that is, we are not disposed to exert ourselves to put the rusting machinery of the law into motion. We must employ some one to do it. What is everybody's business is nobody's. Therefore, let it be some one's especial function to search out and prosecute all violators of the medical practice act wherever they may be found in the State. To bring about this result and to remedy the disease of quackery, the State Medical Association should appoint in every city in Georgia of ten thousand or more inhabitants, a legal representative whose business is to see that the medical law be observed, and whose services shall be paid for by the State Medical Association in the event of the conviction of the offender. The surplus in the treasury is usually between two and three hundred dollars, and is ample to cover this need. Could it be devoted to a better cause? Could it be expended to greater advantage than in clearing the State of quacks and upholding the law which cost so much time and labor to obtain? W.

